# Effect of Time-Restricted Eating versus Daily Calorie Restriction on Mood and Quality of Life in Adults with Obesity

**DOI:** 10.3390/nu15204313

**Published:** 2023-10-10

**Authors:** Shuhao Lin, Sofia Cienfuegos, Mark Ezpeleta, Vasiliki Pavlou, Kaitlin Chakos, Mara McStay, Mary-Claire Runchey, Shaina J. Alexandria, Krista A. Varady

**Affiliations:** 1 Department of Kinesiology and Nutrition, University of Illinois Chicago, 1919 West Taylor Street, Room 532, Chicago, IL 60612, USA; 2 Department of Preventative Medicine (Biostatistics), Northwestern University, Chicago, IL 60612, USA

**Keywords:** time-restricted eating, intermittent fasting, calorie restriction, mood, depression, quality of life, obesity, weight loss

## Abstract

The purpose of this secondary analysis is to compare the effects of two popular weight loss regimens, time-restricted eating (TRE) and daily calorie restriction (CR), on mood and quality-of-life measures in adults with obesity. Ninety participants were randomized to one of three interventions for 12 months: 8 h TRE (eating only between 12:00 and 8:00 p.m., with no calorie counting); CR (25% energy restriction daily); or no-intervention control group. Questionnaires were administered to measure mood (Beck Depression Inventory-II (BDI-II), and Profile of Mood States (POMS)) and quality of life (Rand 36-Item Short Form) at baseline and month 12. Body weight decreased in the TRE group (−4.87%, 95%CI: −7.61, −2.13) and CR group (−5.30%, 95%CI: −9.06, −1.54) versus controls, with no difference between TRE and CR. The BDI-II depression score did not change in the TRE or CR group, versus controls, by month 12. Likewise, there were no changes in any of the POMS subscales (tension, depression, anger, fatigue, anger, confusion, or vigor) or the total mood disturbance score in the TRE or CR group versus controls. As for quality of life, there were no significant changes in the SF-36 constructs of mental health, bodily pain, and general physical health in the TRE or CR group versus controls. However, there was a trend towards increased vitality in the TRE group (7.77 [95% CI: 0.15, 15.39] *p* = 0.05) relative to controls. There were no associations between changes in body weight, physical activity, mood, and quality of life in any group by the end of the study. These findings suggest that TRE and CR produce similar degrees of weight loss, but impact neither mood nor quality of life in adults with obesity over 12 months. Future well-powered studies will be needed to confirm these findings.

## 1. Introduction

Depression and mood disturbances are commonly associated with obesity [1,2]. Evidence suggests that individuals with obesity are 20–50% more likely to meet criteria for major depressive disorders than individuals who are normal weight [1,2]. In those with severe obesity (i.e., BMI greater than 40 kg/m^2^), the risk of mood disorders is even greater [3]. The relationship between mood disturbances and obesity seems to be bi-directional; some observational studies show that depression is associated with subsequent weight gain and obesity [4,5], while other trials demonstrate that obesity is associated with the development of depression [6,7]. Moreover, females with obesity appear to be particularly vulnerable to depression when compared to their male counterparts [8,9].

Obesity is also associated with lower quality of life [10,11]. Health-related quality of life is a concept that examines how a person’s physical and mental health impacts their ability to live a fulfilling life. Individuals with obesity generally report impairments in physical functioning, general health, psychological status, and augmented bodily pain, which can contribute to lower quality of life [12,13]. Evidence also suggests that more severe obesity is associated with greater impairments in quality of life [14,15].

Intentional reductions in body weight have been shown to improve both mood and quality of life [16,17]. Most studies that measure the effect of weight reduction on these variables employ daily calorie restriction protocols [16,17] For instance, in a 2-year study by Martin et al. [18], daily CR (25% energy restriction daily) produced 10% weight loss, accompanied by improvements in mood and general health (a quality-of-life measure), relative to controls. Similarly, Prehn et al. [19] demonstrated a reduction in depression scores with 10% weight loss after 3 months of 35% CR, relative to controls.

In recent years, time-restricted eating (TRE) has gained popularity as a weight loss regimen. TRE involves shortening the eating window to 4–10 h per day and fasting with energy-free beverages for the remaining hours [20]. TRE differs from CR in that it requires individuals to count time, instead of counting calories, to lose weight. Evidence suggests that TRE can lead to mild to moderate weight loss (3–5%) in 2–12 months in adults with obesity [21,22,23,24,25,26,27]. What remains unknown, however, is whether these reductions in body weight through TRE are accompanied by changes in mood and quality of life.

We recently conducted a 12-month randomized controlled trial comparing the effects of 8 h TRE and CR on body weight in adults with obesity [28]. Briefly, we showed that both 8 h TRE (12–8 p.m. eating window) and CR (25% energy restriction daily) resulted in similar reductions in caloric intake (~400 kcal) and body weight (~5%) over one year. The purpose of this secondary analysis is to compare the effects of TRE versus CR on mood and quality of life measures over 12 months. We hypothesized that TRE and CR would produce similar improvements in these measures, relative to controls, as both diet therapies lead to comparable reductions in body weight.

## 2. Methods

### 2.1. Participant Selection and Randomization

This study is a secondary analysis of a previously published 12-month randomized controlled trial [28]. The study compared the effects of 8 h TRE to those of daily CR on body weight and metabolic disease risk factors in adults with obesity. The Office for the Protection of Research Participants at the University of Illinois, Chicago, approved the experimental protocol, and all volunteers gave written informed consent to participate in the trial. Participants were recruited from the Chicago area through posters placed around the University of Illinois Chicago campus.

Inclusion criteria: male or female; age between 18 and 65 years; and BMI between 30 and 50 kg/m^2^. Exclusion criteria: history of diabetes mellitus; history of eating disorders; use of medications that induce weight loss; weight unstable for 3 months prior to the beginning of the study (>4 kg weight loss or gain); eating within less than a 10 h window; perimenopausal; nightshift workers; pregnant or trying to become pregnant; and current smokers. Subjects were screened via BMI assessment, pregnancy test, questionnaire, and a food log. Subjects were randomized using a stratified random sampling protocol (by age, sex, and BMI) into one of three groups: 8 h TRE, 25% CR, or a no-intervention control group. The 12-month trial was divided into a 6-month weight loss phase followed by a 6-month weight maintenance phase. Due to the nature of the interventions, subjects could not be blinded as to their intervention assignment.

### 2.2. Time-Restricted Eating Protocol

During the 6-month weight loss phase, TRE subjects were asked to eat only between the hours of 12:00 and 8:00 p.m. and to fast from 8:00 to 12:00 p.m. the following day. Subjects did not have to monitor food or energy intake during the 8 h eating window. During the 16 h fasting window, subjects were allowed to drink water and calorie-free beverages like coffee and tea without additives, and diet sodas (limit 2 per day). During the 6-month weight maintenance period, subjects were asked to maintain their weight by expanding their eating window to 10:00 a.m. to 8:00 p.m. (10 h eating window) and fast from 8:00 p.m. to 10:00 a.m. (14 h fasting window). TRE subjects received diet counseling (over Zoom) weekly during the first 3 months and then biweekly from months 4 to 12. During the counseling sessions, subjects learned how to make healthy food choices [29] and were also taught cognitive behavioral strategies to assist with controlling weight.

### 2.3. Calorie Restriction Protocol

During the 6-month weight loss phase, CR subjects were asked to reduce their energy intake by 25% each day. Total energy expenditure was calculated using the Mifflin equation [30] and multiplied by the applicable activity factor for each participant. CR subjects met with the dietitian at the commencement of the study to develop weight loss meal plans according to their food preferences. During the 6-month weight maintenance phase, CR subjects were instructed to consume 100% of their newly calculated energy needs. CR subjects received diet counseling (over Zoom) weekly during the first 3 months and then biweekly from months 4 to 12. During the counseling sessions, subjects learned how to make healthy food choices [29] and were also taught cognitive behavioral strategies to assist with weight control.

### 2.4. Control Group Protocol

Control subjects were asked to maintain their weight by not changing their eating and activity habits during the 12-month trial. Controls did not receive diet counseling but were contacted by the study coordinators at the same frequency as the TRE and CR groups to provide body weight measurements.

### 2.5. Assessment of Body Weight and Body Composition

All variables were measured at baseline and month 12. Body weight was assessed without shoes, in light clothing, using a digital scale at the research center. Body composition (fat mass, lean mass, and visceral fat ass) was measured using dual X-ray absorptiometry (iDXA, GE). Waist circumference was measured using a measuring tape midway between the lowest rib and the iliac crest by trained study personnel.

### 2.6. Diet Adherence and Physical Activity

Adherence to the TRE diet was measured using a paper or digital adherence log, which recorded the time each participant started and stopped eating each day. The day was labeled as adherent if the log showed that the subject ate within the correct eating window that day. If they did not, the day was labeled as nonadherent. Compliance with the TRE diet was expressed as percentage of adherent days from baseline to month 12. Adherence to CR was expressed as the percentage of participants who were compliant with their calorie targets (within ±200 kcal). Energy intake and dietary composition were assessed through a 7-day food log using the Automated Self-Administered 24-hour (ASA-24) diet assessment tool [31]. All subjects were instructed not to change their exercise habits during the trial. Physical activity (steps/d) was measured using a pedometer (Fitbit Alta HR) worn continuously for 7 days at baseline and month 12.

### 2.7. Assessment of Mood

Mood was quantified with the Beck Depression Inventory II (BDI-II) [32] and the Profile of Mood States (POMS) [33]. The BDI-II is a reliable and valid measure of mood disturbance, with a score range of 0 to 63, where higher scores indicate worse mood [32]. More specifically, scores of 0–13 are considered no depression, 14–19 is mild depression, 20–28 is moderate depression, and 29–63 is severe depression [32]. The POMS has 6 subscales: tension (score range, 0–36), depression (score range, 0–60), anger (score range, 0–48), fatigue (score range, 0–28), vigor (score range, 0–32), and confusion (score range, 0–28), and a total mood disturbance (score range, −32 to 200) [33]. High vigor scores reflect a good mood or emotion, and low scores in the other subscales reflect a good mood or emotion. The total mood disturbance score is computed by adding the five negative subscale scores (tension, depression, anger, vigor, fatigue, and confusion) and subtracting the vigor score. Higher scores for the total mood disturbance score indicate a greater degree of mood disturbance.

### 2.8. Assessment of Quality of Life

Quality of life was measured using the Rand 36-Item Short Form (SF-36) [34]. The SF-36 has 4 subscales: 2 that measure mental aspects of quality of life (vitality and mental health) and 2 that measure physical aspects of quality of life (bodily pain and physical health). Scores on the SF-36 range from 0 to 100, with higher scores reflecting better quality of life.

### 2.9. Statistical Analyses

Data are shown as mean (95% CI) unless otherwise noted. We conducted an intention-to-treat analysis, which included data from all 90 participants who were randomly assigned. Results are reported for the intention-to-treat analysis unless indicated otherwise. A linear mixed model was used to assess time, group, and time × group effects for each outcome. In each model, time and group effects (and their interaction) were estimated without imposing a linear time trend. For each outcome variable, linear modeling assumptions were assessed with residual diagnostics. To account for the potential for nonuniform variances (heteroskedasticity) between treatment groups due to random chance, all CIs and *p* values from linear mixed models were calculated using robust variance estimators (sandwich estimators). Pearson correlations were performed to assess the relationships between changes in body weight, physical activity, mood, and quality of life. All analyses were performed using R software.

## 3. Results

### 3.1. Baseline Characteristics

As reported previously [28], 126 subjects were screened and 90 were randomized into the TRE group (*n* = 30), CR group (*n* = 30), or control group (*n* = 30). By the end of the 12-month study, the number of dropouts were as follows: TRE group *n* = 4 subjects, CR group *n* = 5 subjects, and control group *n* = 4 subjects. Reasons for dropping out were scheduling conflicts, personal reasons, and inability to contact. All baseline characteristics had similar distributions between the TRE, CR, and control groups (Table 1). The subjects were primarily non-Hispanic Black and Hispanic women with obesity.

### 3.2. Body Weight and Body Composition

Changes in body weight and body composition are reported in Table 2. By month 12, body weight significantly decreased in the TRE group (−4.87% [95% CI: −7.61, −2.13]) and CR group (−5.30% [95% CI: −9.06, −1.54]) versus controls, with no difference between the TRE and CR (0.43% [95% CI: −3.48, 4.34]). Fat mass was reduced in the TRE group (−2.77 kg [95% CI: −5.10, −0.43]) versus controls, but not in the CR group versus controls (−3.18 kg [95% CI: −6.85, 0.49]). Lean mass decreased in the CR group versus controls (−1.13 kg [95% CI: −2.24, −0.01]), but not in the TRE group versus controls (−0.81 kg [95% CI: −1.81, 0.20]). Visceral fat mass did not differ in the TRE and CR groups versus controls by month 12. Waist circumference was reduced in TRE group compared to the control group (−4.98 cm [95% CI: −8.09, −1.87]), but not in the CR group versus controls (−2.30 cm [95% CI: −6.55, 1.94]).

### 3.3. Diet Adherence and Physical Activity

TRE subjects adhered to their eating window on 87% of days on average over the 12-month trial. In the CR group, 61% of subjects adhered to their prescribed calorie targets over 12 months. Energy intake decreased by −425 kcal/d (SD 531 kcal/d) in the TRE group and −405 kcal/d (SD 712 kcal/d) in the CR group, with no difference between groups, over 12 months. As reported previously [28], percent energy intake from protein, carbohydrates, and fat did not change over the course other trial in the TRE and CR groups versus controls. Changes in physical activity (steps per day) did not differ over time or between groups [28].

### 3.4. Mood

**Figure 1:**
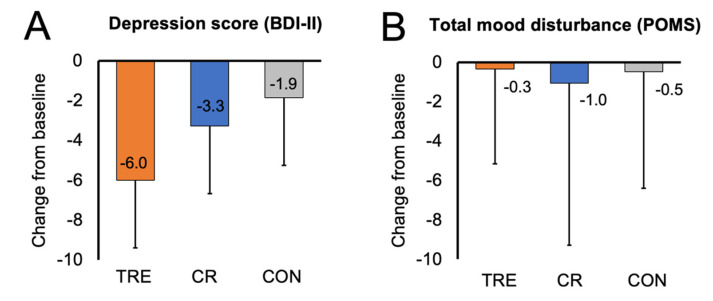
Change in mood scores by month 12. Data are included for 90 participants; means were estimated using an intention-to-treat analysis using a linear mixed model. Error bars indicate 95% confidence intervals for each parameter from baseline by diet group. (**A**) Change from baseline to month 12 in depression score (measured with BDI-II survey). (**B**) Change from baseline to month 12 in total mood disturbance score (measured with SF-36 survey). Abbreviations. BDI-II: Beck Depression Inventory II; CON: control; CR: calorie restriction; POMS: Profile of Mood States survey; TRE: time-restricted eating.

Changes in mood are presented in Table 2 and Figure 1. Results of the BDI-II survey show that the TRE group had mild depression at baseline, while the CR group and control group had no identifiable depression at baseline. The BDI-II depression score did not change in the TRE or CR group, versus controls, by month 12. The POMS tension, depression, anger, fatigue, and confusion scales were low at baseline, while the vigor (good mood) scale was high at baseline in all groups. Moreover, the POMS total mood disturbance score was low in all groups at baseline. Taken together, this would indicate that the participants in each group had a good general mood at the beginning of the study. By month 12, there were no changes in any of the POMS subscales or the total mood disturbance score in the TRE or CR groups versus controls.

### 3.5. Quality of Life

**Figure 2:**
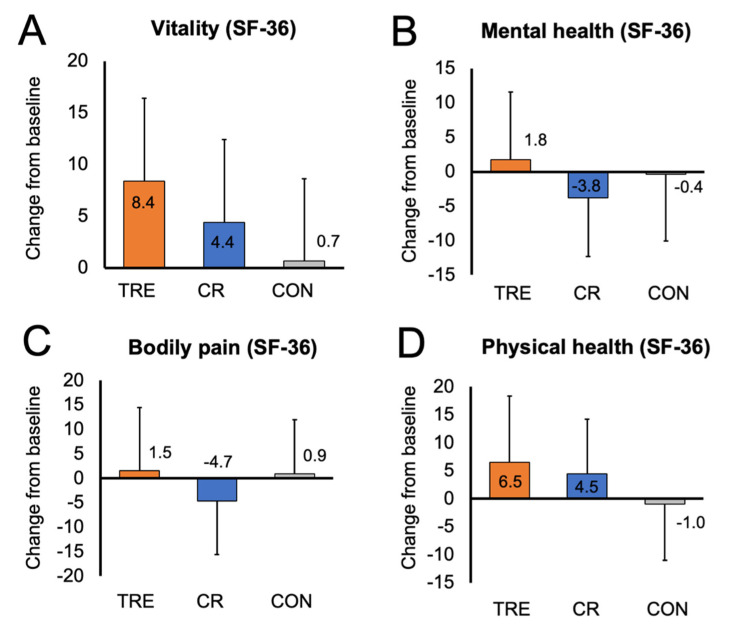
Change in quality of life constructs by month 12. Data are included for 90 participants; means were estimated using an intention-to-treat analysis using a linear mixed model. Error bars indicate 95% confidence intervals for each parameter from baseline by diet group. (**A**) Change from baseline to month 12 in vitality score (measured with SF-36 survey). (**B**) Change from baseline to month 12 in mental health score (measured with SF-36 survey). (**C**) Change from baseline to month 12 in bodily pain score (measured with SF-36 survey). (**D**) Change from baseline to month 12 in general physical health score (measured with SF-36 survey). Abbreviations. CON: control; CR: calorie restriction; SF-36: Rand 36-Item Short Form survey; TRE: time-restricted eating.

Changes in quality of life are presented in Table 2 and Figure 2. At baseline, the SF-36 constructs of vitality, mental health, bodily pain, and general physical health were all moderately high in the TRE, CR, and control groups. This would indicate overall good quality of life in all groups at the beginning of the study. After 12 months of intervention, there were no significant changes in mental health, bodily pain, and general physical health in the TRE or CR groups versus controls. However, there was a trend towards increased vitality in the TRE group (7.77 [95% CI: 0.15, 15.39] *p* = 0.05), relative to controls.

### 3.6. Relationships between Changes in Body Weight, Physical Activity, Mood, and Quality of Life

There were no associations between changes in body weight, physical activity, mood, or quality of life in any group by the end of the study.

## 4. Discussion

To our knowledge, this is the first study to compare the effects of TRE (without calorie counting) versus daily CR on mood and quality of life in adults with obesity. Our results indicate that 12 months of TRE and CR have no effect on mood or quality of life in this population group, relative to no-intervention controls.

Obesity is associated with a higher risk of mood disorders and depression [2,35], Weight loss through dietary interventions has been shown to reduce depression symptoms [36]. The results from our study show that neither TRE nor CR significantly impacted mood in people with obesity. However, our findings are somewhat contradictory to what has been observed previously [18,37,38,39]. For example, in the study by Martin et al. [18], depression symptoms (measured with the BDI-II survey) and tension (measured with the POMS survey) significantly improved after 2 years of CR and 10% weight loss in overweight adults. Moreover, the results of a systematic review by Pastalos et al. [38] demonstrate significant improvements in depression symptoms when 6–10% weight loss is attained with CR.

While several studies have examined the impact of CR on mood, only two studies [37,40] have measured these outcomes for TRE. Fagundes et al. [39] conducted an 8-week study to compare the effects of 8 h TRE versus no-intervention controls. They found that 8 h TRE resulted in a significant decrease in body weight (6%) and stress levels but had no impact on symptoms of depression [39]. Steger et al. [37] compared the effects of early TRE combined with caloric restriction to those of caloric restriction alone on mood. They found that combining early TRE with caloric restriction led to greater weight loss (7%) and improvements in total mood disturbance score (measured by the POMS survey), versus caloric restriction alone (4% weight loss), after 14 weeks. 

In view of these previous findings, it would appear as though at least 6% weight loss is needed to see changes in mood. Since our TRE and CR participants only achieved approximately 5% weight loss, it is possible that the degree of weight reduction was not large enough to see changes in mood scores.

While the exact mechanism linking depression and obesity remains unclear, it is likely that the hypothalamic–pituitary–adrenal (HPA) axis, also known as the reward center of the brain, plays a major role [2,35]. Dysregulation of the HPA axis has been observed in both depression and obesity, which may partially be caused by decreased brain-derived neurotrophic factor (BDNF) [41]. The BDNF pathway plays an important role in neuroplasticity, and it has been shown that increased BDNF levels are associated with reduced symptoms of depression in pre-clinical studies [41,42]. Moreover, recent evidence from human trials shows that weight-reducing diets, such as daily calorie counting, can increase BDNF levels in humans and improve mood [23,43,44,45,46]. Measuring how TRE impacts levels of BDNF and mood will be an important next step in this field.

Individuals with obesity have also been shown to have lower quality of life [17,47]. Obesity is associated with negative social stigma that can lead to lower self-esteem and worsen body image [48]. In addition, obesity is associated with decreased mobility and increased knee pain [49]. Evidence has shown that weight loss can help individuals with obesity regain self-esteem, decrease pain, and improve quality of life [36]. However, improvements in quality of life are generally seen with a weight reduction ranging from 6 to 10% [36]. 

Findings from the present trial suggest that TRE and CR do not significantly impact quality of life in adults with obesity. Our data differ from previous reports, which generally show that TRE and CR have mild positive effects on quality of life. For instance, Carson et al. [50] conducted a systematic review and found significant improvements in quality of life with CR interventions that produced 7–10% weight loss [50]. In line with these findings, Martin et al. [18] demonstrated improvements in the quality of life construct “general health” (measured by the SF-36) in the CR group with 10% weight loss, versus controls. As for TRE, only five studies to date have examined how this intervention impacts quality of life [51,52,53,54,55]. Crose et al. [54] demonstrated significant improvements in the construct “emotional health” (measured by the SF-36 survey) through 8 h TRE with 5% weight loss versus controls. Similarly, Kesztyus et al. [51] and Schroder et al. [52] showed significant improvements in health-related quality of life after 12 weeks of TRE compared to baseline, with 5–6% weight loss. On the other hand, Anton et al. [53] and Parr et al. [55] demonstrated no change in any quality of life parameter with TRE when only 2–3% weight loss was achieved. Thus, similar to mood, it is possible that quality-of-life measures only improve when clinically significant weight loss is achieved (>5% from baseline).

Our study has several limitations. First, our sample size was small (*n* = 90). Thus, it is likely that we were underpowered to detect significant changes in these secondary outcome measures. Second, our study population exhibited low depression and high quality-of-life scores at baseline, which may have hindered our ability to detect improvements in these variables. Third, our study sample was mostly female (~80%), which limits the generalizability of our findings. Lastly, the study used only quantitative data from self-reported questionnaires. The study would have been strengthened by qualitative data from interviews conducted before and after the interventions.

## 5. Conclusions

In conclusion, our findings suggest that TRE and CR produce similar degrees of weight loss, but impact neither mood nor quality of life in adults with obesity over 12 months. However, our participants had healthy mood and quality-of-life scores at baseline, and only achieved a minimal amount of weight loss (5%), which may explain why we observed no effect. Future well-powered studies will be needed to confirm these findings.

## Figures and Tables

**Table 1:** Baseline characteristics of the study participants.

	TRE	CR	Control
***n***	30	30	30
**Age (**year**)**	44 ± 12	44 ± 9	44 ± 13
**Sex, no. (%)**			
Female	25 (83%)	24 (80%)	25 (83%)
Male	5 (17%)	6 (20%)	5 (17%)
**Race or ethnic group, no. (%)**			
Black	11 (37%)	9 (30%)	10 (33%)
Asian	3 (10%)	3 (10%)	0 (0%)
Hispanic	13 (43%)	11 (37%)	17 (57%)
White	3 (10%)	7 (23%)	3 (10%)
**Body weight and composition**			
Body weight (kg)	100 ± 17	102 ± 18	102 ± 17
Fat mass (kg)	46 ± 11	47 ± 11	47 ± 10
Lean mass (kg)	50 ± 10	50 ± 9	51 ± 8
Visceral fat mass (kg)	1.6 ± 0.6	1.6 ± 0.8	1.7 ± 0.8
Waist circumference (cm)	109 ± 13	110 ± 14	110 ± 13
Height (cm)	164 ± 9	166 ± 9	165 ± 7
BMI (kg/m^2^)	37 ± 6	37 ± 5	38 ± 5
**Physical activity (steps/day)**	6465 ± 2653	6627 ± 2842	6424 ± 2302
**Mood**			
BDI-II, depression score	15 ± 8	12 ± 12	10 ± 8
POMS, tension	3 ± 3	2 ± 3	4 ± 5
POMS, depression	1 ± 2	2 ± 5	1 ± 2
POMS, anger	1 ± 2	2 ± 4	1 ± 2
POMS, fatigue	3 ± 3	3 ± 5	3 ± 4
POMS, vigor (good mood)	7 ± 4	8 ± 4	9 ± 5
POMS, confusion	2 ± 3	2 ± 3	2 ± 2
POMS, total mood disturbance score	−14 ± 3	−10 ± 24	−18 ± 14
**Quality of Life**			
SF-36, vitality	54 ± 19	60 ± 21	64 ± 19
SF-36, mental health	74 ± 16	77 ± 19	80 ± 14
SF-36, bodily pain	72 ± 24	81 ± 17	82 ± 24
SF-36, general physical health	62 ± 18	62 ± 23	66 ± 18

Data are expressed as mean ± SD unless otherwise indicated. BMI: body mass index; BDI-II: Beck Depression Inventory II; CR: calorie restriction; POMS: Profile of Mood States; SF-36: Rand 36-Item Short Form; TRE: time-restricted eating.

**Table 2:** Change in body weight, body composition, mood, and quality of life parameters by month 12.

Variables	Change from Baseline to Month 12 (95% CI)			Difference between Groups by Month 12 (95% CI)		
	TRE	CR	CON	TRE vs. CR	TRE vs. CON	CR vs. CON
**Body weight and composition**						
Body weight (kg)	−3.49 (−5.65, −1.32)	−4.30 (−7.63, −0.96)	1.12 (−0.69, 2.94)	0.81 (−3.07, 4.69)	**−4.61 (−7.37, −1.85)**	**−5.42 (−9.13, −1.71)**
Body weight (%)	−3.76 (−5.89, −1.64)	−4.20 (−7.59, −0.80)	1.11 (−0.72, 2.94)	0.43 (−3.48, 4.34)	**−4.87 (−7.61, −2.13)**	**−5.30 (−9.06, −1.54)**
Fat mass (kg)	−2.20 (−3.88, −0.52)	−2.61 (−5.97, 0.74)	0.57 (−1.14, 2.27)	0.42 (−3.24, 4.07)	**−2.77 (−5.10, −0.43)**	−3.18 (−6.85, 0.49)
Lean mass (kg)	−0.41 (−0.91, 0.08)	−0.74 (−1.44, −0.03)	0.39 (−0.51, 1.29)	0.32 (−0.52, 1.16)	−0.81 (−1.81, 0.20)	**−1.13 (−2.24, −0.01)**
Visceral fat mass (kg)	−0.14 (−0.23, −0.04)	−0.12 (−0.29, 0.06)	−0.03 (−0.16, 0.10)	−0.02 (−0.22, 0.17)	−0.11 (−0.27, 0.06)	−0.08 (−0.30, 0.13)
Waist circumference (cm)	−6.44 (−8.65, −4.24)	−3.77 (−7.46, −0.08)	−1.46 (−3.77, 0.84)	−2.67 (−6.86, 1.52)	**−4.98 (−8.09, −1.87)**	−2.30 (−6.55, 1.94)
**Mood**						
BDI-II, depression score	−5.99 (−9.37, −2.60)	−3.27 (−6.55, 0.01)	−1.85 (−5.26, 1.55)	−2.72 (−7.32, 1.88)	−4.13 (−8.82, 0.56)	−1.41 (−6.03, 3.20)
POMS, tension	0.02 (−1.31, 1.35)	−0.35 (−2.19, 1.49)	0.19 (−1.12, 1.50)	0.37 (−1.85, 2.59)	−0.16 (−1.99, 1.66)	−0.53 (−2.74, 1.67)
POMS, depression	0.84 (−0.05, 1.72)	0.53 (−1.09, 2.16)	0.12 (−0.82, 1.06)	0.31 (−1.50, 2.11)	0.72 (−0.54, 1.98)	0.41 (−1.42, 2.25)
POMS, anger	0.39 (−0.47, 1.24)	0.56 (−1.29, 2.41)	−0.38 (−1.37, 0.60)	−0.17 (−2.16, 1.82)	0.77 (−0.50, 2.05)	0.94 (−1.10, 2.99)
POMS, fatigue	0.68 (−0.58, 1.95)	−0.28 (−1.61, 1.04)	0.41 (−0.91, 1.73)	0.97 (−0.82, 2.75)	0.27 (−1.51, 2.06)	−0.69 (−2.52, 1.13)
POMS, vigor (good mood)	0.86 (−0.98, 2.69)	1.19 (−0.52, 2.90)	0.26 (−1.29, 1.81)	−0.33 (−2.79, 2.12)	0.60 (−1.75, 2.94)	0.93 (−1.32, 3.18)
POMS, confusion	−0.22 (−1.30, 0.87)	−0.48 (−1.75, 0.78)	−0.20 (−1.35, 0.95)	0.27 (−1.36, 1.89)	−0.02 (−1.56, 1.52)	−0.29 (−1.95, 1.38)
POMS, total mood disturbance score	−0.33 (−5.15, 4.49)	−1.05 (−9.29, 7.19)	−0.47 (−6.40, 5.46)	0.72 (−8.59, 10.03)	0.14 (−7.32, 7.60)	−0.58 (−10.49, 9.32)
**Quality of Life**						
SF-36, vitality	8.42 (2.86, 13.98)	4.42 (−2.63, 11.47)	0.65 (−4.82, 6.12)	4.00 (−4.76, 12.76)	7.77 (0.15, 15.39), *p* = 0.05	3.77 (−4.93, 12.47)
SF-36, mental health	1.77 (−4.41, 7.95)	−3.79 (−11.65, 4.07)	−0.36 (−6.46, 5.75)	5.56 (−4.20, 15.32)	2.13 (−6.36, 10.61)	−3.43 (−13.14, 6.28)
SF-36, bodily pain	1.52 (−7.72, 10.75)	−4.68 (−14.20, 4.84)	0.90 (−5.45, 7.25)	6.20 (−6.75, 19.14)	0.61 (−10.33, 11.55)	−5.58 (−16.75, 5.58)
SF-36, general physical health	6.53 (−1.80, 14.86)	4.46 (−4.25, 13.17)	−0.96 (−6.60, 4.68)	2.07 (−9.68, 13.83)	7.49 (−2.32, 17.30)	5.42 (−4.71, 15.55)

Data were included for 90 participants; means were estimated using an intention-to-treat analysis using a linear mixed model. Error bars indicate 95% confidence intervals for each parameter from baseline by diet group. Abbreviations: BDI-II: Beck Depression Inventory II; CON: control; CR: calorie restriction; POMS: Profile of Mood States; SF-36: Rand 36-Item Short Form; TRE: time-restricted eating.

## Data Availability

Data are unavailable due to privacy or ethical restrictions.

## Trial Registration

Clinicaltrials.gov, NCT04692532.
